# The role of the caudate nucleus in taste function: a comprehensive narrative review

**DOI:** 10.3389/fnins.2026.1783785

**Published:** 2026-05-20

**Authors:** Maria Jimena Ricatti, Federico Boschi, Silvia Savazzi, Gianluigi Zanusso, Maria Paola Cecchini

**Affiliations:** 1 Anatomy and Histology Section, Department of Neurosciences, Biomedicine and Movement Sciences, University of Verona, Verona, Italy; 2 Engineering and Physics Section, Department of Engineering for Innovation Medicine, University of Verona, Verona, Italy; 3 Perception and Awareness (PandA) Lab, Department of Neurosciences, Biomedicine and Movement Sciences, University of Verona, Verona, Italy; 4 Neurology Unit, Department of Neurosciences, Biomedicine and Movement Sciences, University of Verona, Verona, Italy

**Keywords:** caudate nucleus, feeding behavior, neuroimaging, reward system, taste function

## Abstract

The caudate nucleus, a key component of the dorsal striatum, has traditionally been recognized for its roles in motor control and cognitive functions. However, emerging neuroimaging and neurophysiological findings show its crucial involvement in gustatory function as well. The papers analyzed in this comprehensive overview indicate that the caudate nucleus and dorsal striatum exhibit consistent activation during gustatory stimulation, respond to different metabolic states, motivation, and hedonic value, and interact with regions involved in reward and emotional processing across health and disease. Even if these results are promising, experimental designs are frequently heterogeneous, so more evidence is needed to elucidate the link between taste and these subcortical regions. This approach may provide new perspectives on the neural substrates of chemosensation and potential targets for taste-related interventions.

## Introduction

The caudate nucleus, a major component of the dorsal striatum, historically associated with motor and cognitive functions (Haber, 2016; Grahn et al., 2008), has also been implicated in playing a crucial role in various higher-order sensory processing (Kinomura et al., 1994; Savic et al., 2000).

Scientific evidence suggests that this subcortical structure contributes to the integration of multisensory information (Nagy et al., 2006; Mastinu et al., 2025), playing a role in taste and olfactory perception and reward (Jacobson et al., 2010; Rolls, 2019, 2023). The involvement of the caudate nucleus in chemosensory processing, particularly in taste perception, was first demonstrated in the pioneering neuroimaging study by Kinomura and colleagues (Kinomura et al., 1994).

The processing of gustatory stimuli, given its evolutionary importance, involves intricate neural circuits spanning from peripheral receptors to higher cortical areas, allowing key functions that guide food selection, detect environmental hazards, and more (Olofsson and Freiherr, 2019). In addition, these stimuli can elicit innate emotions of pleasure (sweet) and disgust (bitter), hard-wired from birth, in complex interplay with the other senses (Shepherd, 2006).

However, while significant research has mainly focused on cortical mechanisms underlying chemosensory function, the role of subcortical structures, particularly the caudate nucleus, remains less well understood. This review aims to examine the research investigating the role of the caudate nucleus in human taste function, integrating findings from functional and structural imaging studies. We investigate the role of the caudate in the processing of gustatory information and its clinical implications. By elucidating these mechanisms, we aim to provide a comprehensive understanding of the caudate's function in shaping taste perception.

### Anatomy and function of the caudate nucleus

**Figure 1:**
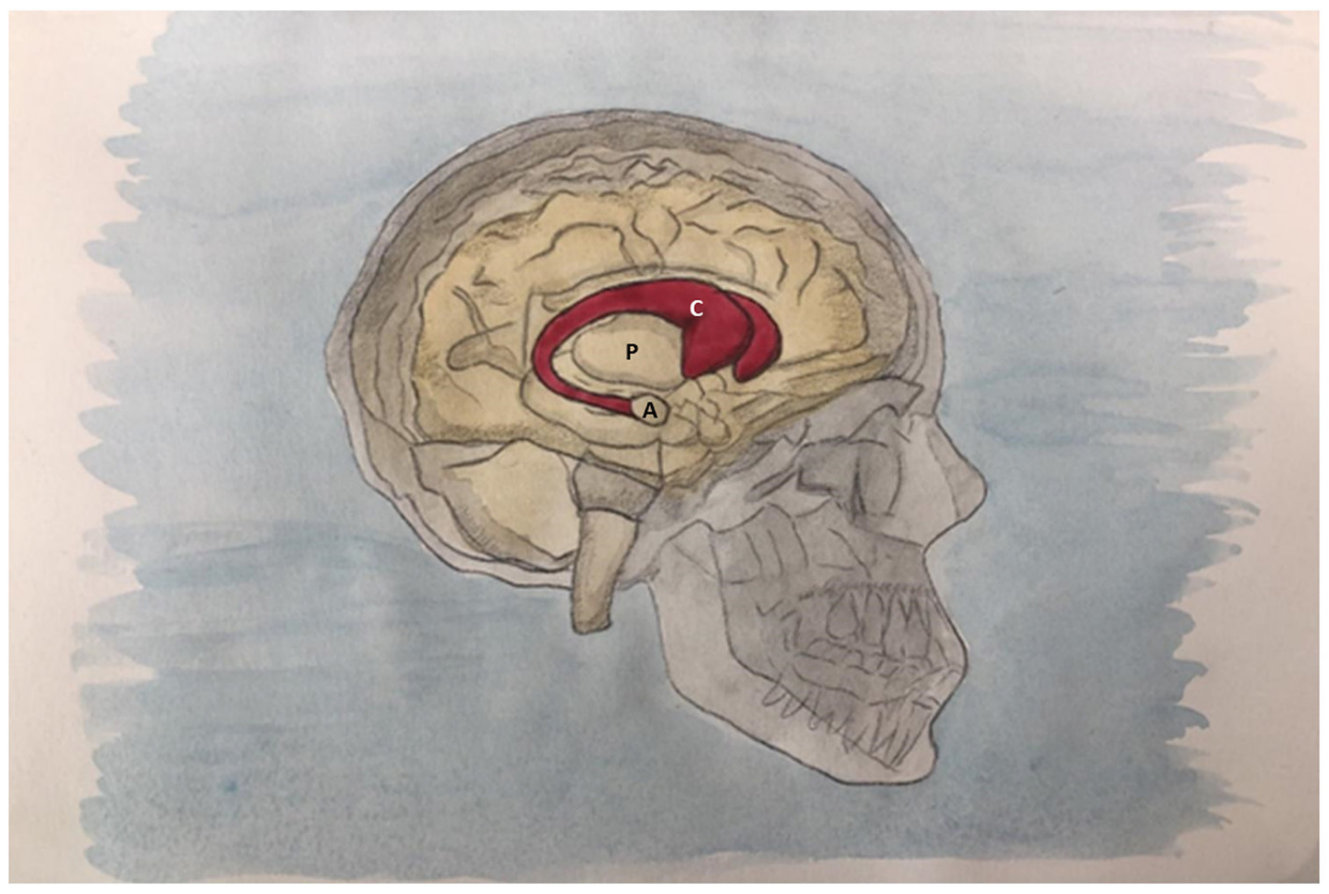
Right side view of the skull and encephalon. In red, a side projection of the caudate nucleus (C), surmounting the thalamus, lying beside the lateral ventricles, with the classical “C” shape, gradually decreasing in volume. Its head extends backwards, with a following body and a tail which curves downwards and laterally to finally end at the level of the temporal pole, in contiguity with the amygdala (A). It belongs to the dorsal striatum along with the putamen (P). During its course, the caudate nucleus is connected to the putamen (P) by means of bridges of gray matter.

The caudate nucleus is a C-shaped subcortical paired structure that constitutes a major part of the dorsal striatum, along with the putamen (Driscoll et al., 2023). It is located sidewise to the lateral ventricles, and each caudate nucleus is divided into three main regions: the head, the body, and the tail (Figure 1). Some studies in humans and animals suggest that these regions play different functional roles, with the caudate head contributing to goal-directed planning and sub-goal selection by evaluating action-outcomes (Grahn et al., 2008, 2009), whereas the caudate tail appears to be more involved in processing sensory inputs (Saint-Cyr et al., 1990; Yeterian and Pandya, 1995; Griggs et al., 2017). However, the anatomo-functional correlation among the caudate regions remains under study, as there is mixed evidence regarding this topographic organization, with some research suggesting that these areas may rapidly update the value of stimuli based on reward, indicating a more flexible function in terms of information processing, rather than strictly categorical manner (Kim et al., 2014). In addition, brain lateralization has been documented across the central nervous system, and the caudate nucleus is no exception (Walker, 1980; Glick et al., 1982). In the context of chemosensory processing, lateralization effects have been observed in the integration of afferent stimuli with higher-order cognitive functions (Royet and Plailly, 2004). Besides, the functional specialization of the caudate nucleus is defined by a complex topographical and biochemical heterogeneity. Detailed *post-mortem* investigation of the human striatum has documented precise gradients of neurotransmitter markers, showing that the highest dopamine (DA) levels increase from rostral to caudal in the caudate and putamen, with the highest concentrations centrally and lowest DA turnover caudally. These results demonstrate a marked heterogeneity in the anatomical distribution of neurotransmitter markers in the human dorsal striatum indicating anatomical and functional diversity within this brain structure (Hörtnagl et al., 2020; De Deurwaerdere et al., 2020). Furthermore, molecular markers related to the protein homeostasis, and synaptic plasticity show specific pathological alterations within the caudate nucleus (Saries-Serrano et al., 2025).

While neurotransmitter gradients are well-documented across the rostro-caudal and dorso-ventral axes in humans, providing a basis for functional diversity, animal research remains more comprehensive in mapping specific genetic markers and circuit-level connectivity (Calipari et al., 2012).

### Anatomy of taste

The taste system can detect and identify the components of ingested food, providing information on the quality of tastants as well as the palatability of food, which allows to distinguish between edible and non-edible ones. The taste information is then conveyed by the cranial nerves (i.e., facial, glossopharyngeal, and vagus) to the central nervous system. Additionally, somatosensory information, such as touch and temperature, contributes together with olfactory and visual stimuli to give the complex flavor experience (Shepherd, 2006; Cecchini et al., 2015).

**Figure 2:**
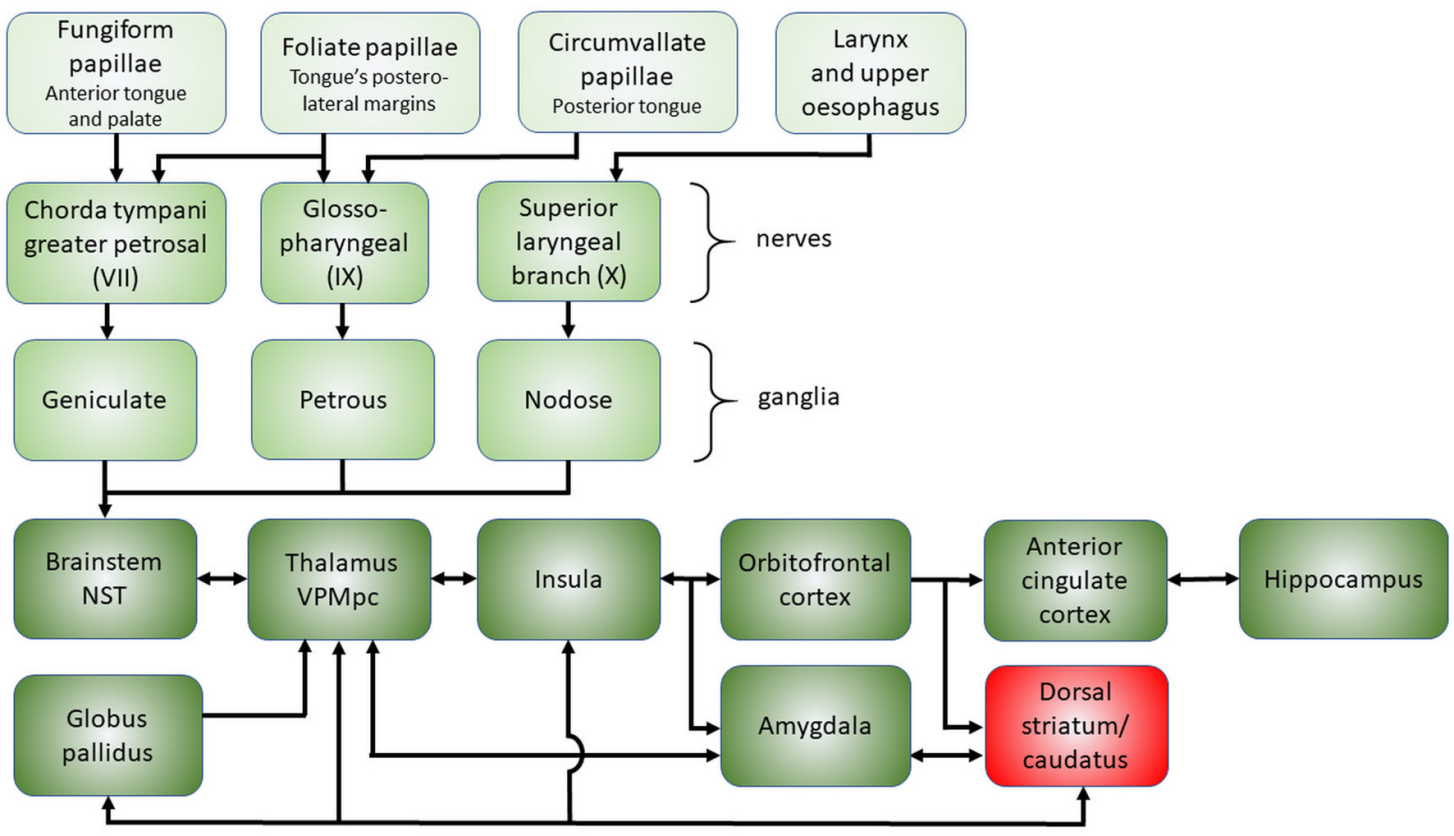
The anatomy of gustatory perception from the peripheral to the more central regions. At the top, the wide peripheral area from which the gustatory stimuli arise (tongue, larynx and esophagus), while at the bottom, the various more central regions involved, including the subcortical nuclei (dorsal striatum/caudatus, red box). The main connections are shown by arrows. The complex network of connections highlights the possible role of the caudate nucleus as a bridge between sensory perception and motor decision-making plans. It could act as a critical hub of the striatum linking basic taste information and motivated behavior. NST, nucleus of the solitary tract; VPMpc, ventral postero-medial nucleus of the thalamus, parvicellular part.

The anatomy of taste starts from a wide mucosal surface, including the whole oral cavity, pharynx, larynx, and upper esophagus (Witt, 2019; Doty, 2019). Unlike olfaction, taste is mediated by several cranial nerves and has receptors widely distributed throughout the oral cavity, which is why it is considered a robust sense (Bartoshuk, 1989; Cecchini et al., 2018). From here, taste message is first transmitted via cranial nerves to the gustatory nucleus, the rostral division of the nucleus of the solitary tract (NST) in the medulla oblongata and pons. Then, NST fibers project to the thalamus (i.e. ventral posteromedial nucleus parvocellular part, VPMpc). Thereupon, thalamic neurons project to the primary taste cortex (e.g., frontal operculum, insula) (Iannilli et al., 2014; Iannilli and Gudziol, 2019). In particular, the anterior insula is implicated in gustatory identification processes, and the posterior insula contributes to oral somatic sensation. (Veldhuizen et al., 2011; Rolls, 2016). Indeed, the insular cortex poses what the taste is, regardless of the linked reward valence. Then, gustatory information reaches the multimodal orbitofrontal cortex, the human key-brain region involved in the subjective emotional experience of the stimuli (Rolls, 2023; Shepherd, 2006). Also amygdala receives gustatory information from the insula, but in humans, comparing to the orbitofrontal cortex, recent evidence show that amygdala is less involved in subjective emotional experience and it is proposed that it is mainly involved in autonomic and conditioned responses trough brainstem connectivity (Rolls, 2023). In addition, the hippocampus connections can have a crucial role in integrating information derived from multiple sensory and cognitive inputs impacting memory (Strauch and Manahan-Vaughan, 2020). Other subcortical areas such as hypothalamus and basal nuclei are involved in taste function, and tractography investigation showed connections between the caudate nucleus and the insular cortex in healthy subjects (Ghaziri et al., 2018), but in this regard current knowledge in humans is not fully elucidated. In particular, gustatory information can reach the striatum through both direct (through insula-striatal projections) and indirect pathways (through connections involving orbitofrontal cortex, amygdala, thalamus). Orbitofrontal cortex's outputs directed to striatum are meaningful enabling the reward value influencing behavior. The reward value outcome information projected to the anterior cingulate cortex it is suggested to be useful for action-outcome learning (Rolls, 2023). On this matter, it is important to mention that taste together with smell have a meaningful role in the expression of hedonic value, represented in various brain areas and this has an important psychological impact (Bochicchio and Winsler, 2020). Moreover, cortical taste regions send efferent projections to the NST and other subcortical areas for top-down gustatory afferent modulation. These projections from the cortex to the NST modulate both the sensory input inside the NST and its efferent output, in integration with olfactory and somatosensory signals (Simon et al., 2006; Iannilli and Gudziol, 2019; Vincis and Fontanini, 2019) (Figure 2).

## Methods

An extensive search of scientific publications on PubMed and Scopus was performed to identify studies examining the role of the caudate nucleus in the different facets of taste function. Given the multifaceted nature of this topic, the search strategy was divided into thematic sections to ensure a comprehensive selection of relevant articles.

### Search strategy

Search was conducted using the following term combinations:

1. Taste and caudate nucleus: (“taste”[MeSH Terms] OR “taste”[All Fields] OR “tastes”[All Fields] OR “tasting”[All Fields] OR “tasted”[All Fields] OR “tasteful”[All Fields] OR “tastings”[All Fields]) AND (“caudate”[All Fields] OR “caudate's”[All Fields] OR “caudates”[All Fields]).
2. Gustation and caudate nucleus: (“taste”[MeSH Terms] OR “taste”[All Fields] OR “gustation”[All Fields]) AND (“caudate nucleus”[MeSH Terms] OR (“caudate”[All Fields] AND “nucleus”[All Fields]) OR “caudate nucleus”[All Fields]).
3. Taste and dorsal striatum: (“taste”[MeSH Terms] OR “taste”[All Fields] OR “tastes”[All Fields] OR “tasting”[All Fields] OR “tasted”[All Fields] OR “tasteful”[All Fields] OR “tastings”[All Fields]) AND (“dorsal”[All Fields] OR “dorsale”[All Fields] OR “dorsally”[All Fields] OR “dorsals”[All Fields]) AND (”neostriatum”[MeSH Terms] OR “neostriatum”[All Fields] OR “striatum”[All Fields] OR “corpus striatum”[MeSH Terms] OR (“corpus”[All Fields] AND “striatum”[All Fields]) OR “corpus striatum”[All Fields] OR “striatum's”[All Fields] OR “striatums”[All Fields]).
4. Gustation and dorsal striatum: (“taste”[MeSH Terms] OR “taste”[All Fields] OR “gustation”[All Fields]) AND (”dorsal”[All Fields] OR “dorsale”[All Fields] OR “dorsally”[All Fields] OR “dorsals”[All Fields]) AND (“neostriatum”[MeSH Terms] OR “neostriatum”[All Fields] OR “striatum”[All Fields] OR “corpus striatum”[MeSH Terms] OR (”corpus”[All Fields] AND “striatum”[All Fields]) OR “corpus striatum”[All Fields] OR “striatum's”[All Fields] OR “striatums”[All Fields]). A similar search strategy was applied in Scopus, using the corresponding free-text terms and Boolean operators.

Inclusion criteria were:

- Peer-reviewed studies published in English.
- Studies investigating the caudate nucleus in relation to taste function in adult humans (psychophysical, neurophysiological, and neuroimaging; either clinical populations or healthy participants).
- Experimental and clinical studies, including neuroimaging research (fMRI, PET, SPECT).

Exclusion criteria were:

- Peer-reviewed studies in languages other than English.
- Studies focused solely on motor or general cognitive functions of the caudate nucleus without reference to taste function.
- Studies on sensory processing without specific mention of the caudate nucleus, dorsal striatum or the basal ganglia.
- Animal studies (unless findings were directly translatable to human function).

## Results

Given the heterogeneity of methodologies across studies, which made comparison difficult, a narrative review approach was chosen and findings across the literature were synthesized. The selected papers are summarized in Table 1 (healthy participants) and Table 2 (pathological conditions) and are discussed separately for the healthy and pathological populations and the caudate's functions.

**Table 1:** Research on healthy participants.

Reference	Neuroimaging method	Sample size gender M (male) F (female)	Stimuli	Stimulation mode	Main result
Perception and discrimination of pure taste stimuli					
Kinomura et al. (1994)	PET	10 M	Salty solution tasting	Ingestion through plastic tubes	Activation of the head of the caudate nucleus of the right hemisphere
Hwang et al. (2019)	fMRI	351 F 208 M	Sweet and bitter solutions	Sipping	Bilateral caudate volume was associated with responses to both sweet and bitter tastes
Avery et al. (2020)	fMRI	11 F 7 M	Sweet, salty, sour, and tasteless solutions	Ingestion through gustometer	The left dorsal striatum and the right caudate discriminate between sweet, salty, and sour tastants
Ponticorvo et al. (2022)	fMRI	25 M 19 F	Sweet and bitter solutions	Ingestion through gustometer	Sweet taste selectively activated the caudate nucleus
Pak et al. (2022)	fMRI	20 M	Sweet solutions	Sipping and Intravenous	Sweet taste selectively activated the caudate nucleus
Reward, anticipation and/or motivational component					
Small et al. (2001)	PET	5 F 4 M	Chocolate tasting	Ingestion	Cerebral blood flow was correlated with pleasantness in the left and right dorsal caudate nucleus
Small et al. (2003)	PET	15 F 2 M	Favorite meal tasting	Ingestion	Dorsal caudate blood flow was correlated with dopamine release and experienced pleasure
Stice et al. (2013)	fMRI	59 F 47 M	Chocolate milkshakes and tasteless solution	Ingestion through gustometer	Fat caused greater activation of the caudate and oral somatosensory regions than sugar
Frankort et al. (2015)	fMRI	34 F	Chocolate tasting	Ingestion	The activation of the right caudate correlated positively with neural predictors of chocolate intake
Blechert et al. (2016)	fMRI	16 F 16 M	Visual stimulation	Food images	The left caudate nucleus showed stronger activation in response to available foods, particularly high-calorie ones
Stice and Yokum (2018)	fMRI	29 F 11 M	High-sugar milkshake stimulation	Ingestion	High-sugar food increases reward response in the right caudate to anticipated intake of more of the high-sugar food
Davidenko et al. (2018)	fMRI	43 M	Sweet-flavored spring water	Ingestion through gustometer	Greater right caudate activation was observed in individuals whose taste judgments were not influenced by conflicting sensory cues
Thanarajah et al. (2019)	fMRI/PET	12 M	Flavored milkshake	Ingestion through gustometer	Delayed dopamine release in the right caudate head was observed post-ingestion
Frank-Podlech et al. (2019)	fMRI	11 M	High- vs. a low-fat yogurt	Ingestion	Post-ingestion connectivity between the caudate nucleus and the nucleus tractus solitarii was reduced regardless of fat content
Lender et al. (2020)	fMRI	11 F 12 M	Marzipan tasting	Ingestion	The left caudate showed increased activation in response to images associated with edible stimuli
Skałbania et al. (2025)	fMRI	49 subjects final gender not specified	Visual stimulation	Food images	The pre-choice activity in the caudate nucleus may potentially exert an influence on linking reward anticipation
Food availability and homeostatic modulation					
Gautier et al. (1999)	PET/MRI	11 M	Sweet flavored liquid meal	Ingestion through plastic tubes	Caudate nucleus is involved in the perception of taste after 36 h of fasting
Pelchat et al. (2004)	fMRI	5 F 5 M	Visual stimulation	Food images	In craving-induced conditions, the right caudate nucleus exhibited increased fMRI activation
Jacobson et al. (2010)	fMRI	30 young adults 22 older adults gender not specified	Salty, sour, bitter, sweet solutions	Ingestion through syringe pumps	Caudate activation in response to gustatory stimuli during hunger
Haase et al. (2011)	fMRI	12 F 9 M	Salty, sour, bitter, sweet solutions	Ingestion through syringe pumps	Bilateral caudate activation was strongly modulated by hunger and satiety when processing reward-related aspects
Skałbania et al. (2025)	fMRI	49 subjects gender not specified	Visual stimulation	Food images	Greater pre-choice caudate activity was linked to higher likelihood of self-control failure in a later food-choice task
Individual features and risk factors					
Jacobson et al. (2010)	fMRI	30 young adults 22 older adults gender not specified	Salty, sour, bitter, sweet solutions	Ingestion through syringe pumps	Older adults exhibited caudate activation in response to gustatory stimuli under motivationally relevant conditions
Haase et al. (2011)	fMRI	12 F 9 M	Salty, sour, bitter, sweet solutions	Ingestion through syringe pumps	Reward processing of some tastants was more strongly modulated by hunger and satiety in males with bilateral caudate activation
Green and Murphy (2012)	fMRI	24 young adults Gender not specified	Sweet solutions tasting	Ingestion through syringe pumps	Individuals who consumed a greater number of diet sodas had reduced right caudate head activation
Green et al. (2013)	fMRI	6 F 6 M	Sweet and bitter solutions tasting	Ingestion through syringe pumps	Caudate showed greater activation in young relative to middle-aged
Bohon (2014)	fMRI	82 F 80 M	Chocolate milkshake	Ingestion through syringe pumps	Emotional eating scores were negatively related to activation in the bilateral caudate
Sun et al. (2015)	fMRI	17 F 15 M	Milkshake consumption	Ingestion through gustometer	In individuals carrying the at-risk allele, caudate activation was associated with long-term weight gain
Horndasch et al. (2016)	fMRI	15 AN family history 21 controls gender not specified	Chocolate drink consumption	Ingestion through syringe pumps	Individuals with a family history of eating disorders showed increased caudate activation during anticipation of food stimuli
Gao et al. (2018)	fMRI	75 F	Visual stimulation	Food images	Greater impulsivity in food-related decisions was linked to reduced right dorsal caudate activity and connectivity
Shearrer et al. (2018)	fMRI	55 F 53 M	Milkshake consumption	Ingestion through gustometer	Familial obesity risk was linked to heightened caudate response to palatable tastes
Sadler et al. (2023)	fMRI	78 F 76 M	Milkshake consumption	Ingestion through syringe pumps	In healthy-weight adolescents, repeated exposure to palatable food cues led to increased caudate activation over time
Pharmacological modulation of taste processing					
Horder et al. (2010)	fMRI	16 F 9 M	Strawberry and chocolate tastants	Ingestion through manual syringe delivery	Rimonabant decreased neural responses to aversive stimulus in the right caudate nucleus
Murray et al. (2014)	fMRI	10 F 10 M	Milkshake consumption	Ingestion through manual syringe delivery	Relative to placebo, naltrexone decreased reward activation to chocolate in the caudate
Tudge et al. (2014)	fMRI	10 F 10 M	Strawberry and chocolate tastants	Ingestion through manual syringe delivery	Tetrahydrocannabivarin increased responses to aversive and rewarding stimuli in the caudate
Dean et al. (2016)	fMRI	9 F 8 M	Belgian chocolate drink tasting	Ingestion through manual syringe delivery	Bupropion increased activity during the anticipation phase of pleasant and unpleasant cues in the caudate

**Table 2:** Research on pathological conditions.

Reference	Neuroimaging method	Sample size gender M (male) F (female)	Stimuli	Stimulation mode	Main result
Obesity and metabolic dysfunction					
Rothemund et al. (2007)	fMRI	13 obese F 13 controls F	Visual stimulation	Food images	The left caudate body showed increased activation in individuals with obesity, independently of hunger or satiation states
Stice et al. (2008b)	fMRI	33 F	Flavored milkshake	Ingestion through syringe pumps	Female obese adolescents showed reduced activation of the caudate nucleus during milkshake consumption
Stice et al. (2008a)	fMRI	43 F (Study 1) 33 F (Study 2)	Flavored milkshake	Ingestion through syringe pumps	Caudate nucleus activation predicted less future weight gain in A1 allele carriers but greater weight gain in non-carriers
Green et al. (2011)	fMRI	20 older adults 20 young adults Gender not specified	Tastants solutions	Ingestion through syringe pumps	In older adults, higher abdominal fat and BMI were associated with reduced caudate activation in response to sucrose
Nummenmaa et al. (2012)	fMRI	19 morbidly obese 16 controls Gender not specified	Visual stimulation	Food images	Enhanced sensitivity to external food cues in obesity may involve abnormal stimulus-response mediated by the dorsal caudate nucleus
Babbs et al. (2013)	fMRI	13 overweight 12 controls Gender not specified	Sweet flavored milkshake	Ingestion through syringe pumps	There is an inverse correlation between BMI and caudate response to milkshakes consumption
Cornier et al. (2015)	fMRI	12 M 12 F Obesity resistant 13 M 12 F Obesity prone	Sucrose solution tasting	Sipping	Neuronal responses to sucrose in the caudate were attenuated in women compared to men
Geha et al. (2017)	fMRI	Tasting 15 controls 15 obese Not-tasting 33 controls 28 obese	Milkshake consumption	Ingestion through oral bolus	Obese individuals showed reduced global brain connectivity in the caudate nucleus both at rest and during milkshake consumption
Jacobson et al. (2017)	fMRI	30 young adults 22 older adults Gender not specified	Sweet tastants	Ingestion through syringe pumps	Reduced caudate activation during sucrose hedonic evaluation under hunger in older adults and metabolic syndrome participants
Dunn et al. (2023)	fMRI	44 F 34 M Gender not specified	Milkshake consumption	Ingestion through syringe pumps	Caudate changes during weight loss and improved insulin sensitivity were less robust than other taste-related striatal responses
Eating disorders					
Wagner et al. (2008)	fMRI	16 F AN recovered 16 F controls	Sweet tastants	Ingestion through syringe pumps	AN recovered participants showed reduced activation in the middle and dorsal caudate in response to nutrient taste
Cowdrey et al. (2011)	fMRI	15 F AN recovered 16 F controls	Flavored solutions	Ingestion through syringe	Individuals recovered from anorexia nervosa showed increased caudate activation in response to aversive taste stimuli
Bohon and Stice (2012)	fMRI	13 F BN 13 F controls	Flavored milkshake	Ingestion	In females with BN, higher negative affect was associated with increased caudate activation during anticipation of a palatable taste
Frank et al. (2013)	MRI	19 F BN 19 F AN 24 F AN all recovered 24 F controls	Sucrose solution	Sipping	Both BN and AN recovered were associated with reduced dorsal caudate gray matter volume
Frank et al. (2018)	fMRI	56 F AN	Sucrose solution	Ingestion through syringe pumps	Individuals with AN showed hyperactivation in the caudate head compared to controls during a dopamine-related conditioning task
Kaye et al. (2020)	fMRI	26 F AN 22 F controls	Sucrose solution and water	Sipping	Lower caudate activation to tastants during hunger was associated with higher harm avoidance in individuals recovered from AN
Frank et al. (2022)	fMRI	28 AN female	Sucrose solution	Ingestion through syringe pumps	At baseline, individuals with AN showed elevated prediction error responses in the bilateral caudate head
Frank et al. (2023)	fMRI	91 AN, 34 other eating disorders, 56 BN, 16 binge eating disorders, 120 controls, all female	Sucrose solution	Ingestion	In eating disorders, greater caudate head response to sucrose receipt was associated with higher reward responsiveness
Stice et al. (2025)	fMRI	88 F	Chocolate milkshake stimulation	Ingestion	The right caudate, a reward valuation region, showed lower responsivity to anticipated milkshake tastes
Alcoholism					
Wilcox et al. (2013)	fMRI	225 M 101 F All heavy drinkers	Alcoholic and non-alcoholic beverages	Ingestion through gustometer	Genetic variation near the α-synuclein gene was significantly associated with caudate activation in heavy drinkers
Ray et al. (2014)	fMRI	11 M 6 F All with alcohol dependence	Alcohol and water taste stimuli	Ingestion through gustometer	In the dorsal caudate, heavy drinkers with the G allele showed stronger negative functional connectivity with other key regions
Monnig et al. (2014)	fMRI	229 M 103 F All heavy drinkers	Alcohol taste cue	Ingestion	In heavy drinkers, reduced white matter integrity was associated with increased caudate activation during alcohol cue exposure
Bidwell et al. (2019)	fMRI	238 M 145 F All hazardous drinkers	Alcoholic and non-alcoholic beverages	Ingestion	In heavy drinkers, higher dopamine receptor D2 methylation was associated with increased caudate activation to alcohol cues
Depression					
McCabe et al. (2009)	fMRI	13 recovered depressed (3 M) 14 controls (5 M)	Flavored tastants	Ingestion through tubes	Recovered depressed individuals showed increased bilateral caudate activation in response to aversive food-related stimuli
COVID-19					
Schönegger et al. (2020)	MRI	5 F	Tastants stimulation	Taste strips	One COVID-19 patient showed a symmetric, slightly hyperintense signal in the head of the caudate nucleus
Cecchini et al. (2024)	fMRI	20 with referred post-Covid-19 smell/taste impairment (6 M) 19 without (11 M)	No gustatory stimulation		A volumetric difference in the right caudate emerged between groups, although it did not remain significant after correction

### Research on healthy participants

#### Perception and discrimination of pure taste stimuli

The involvement of the caudate nucleus in gustatory perception and discrimination has been observed consistently across various studies in healthy participants. This section examines studies utilizing relatively pure tastant-based paradigms, where neural activity is elicited by the chemical properties and intensity of stimuli rather than their associated reward value or anticipatory cues.

Early evidence from Kinomura et al. (1994) demonstrated caudate activation in response to gustatory stimuli, marking one of the first functional neuroimaging reports implicating subcortical structures beyond the brainstem and thalamus in the human taste network. In this study, the head of the caudate nucleus of the right hemisphere was activated, suggesting a potential integrative role in early taste perception.

More recent studies have consistently identified the caudate nucleus as a key region involved in gustatory perception. In 2019, Hwang et al. further expanded knowledge on these observations by showing that the volume of the bilateral caudate was associated with sensitivity to both sweet and bitter taste stimuli. Authors suggest that the caudate nucleus is involved structurally in processing both taste qualities.

Moreover, in a multivoxel pattern analysis study, Avery et al. (2020) demonstrated that the left dorsal striatum and the right caudate could reliably discriminate between sweet, salty, and sour tastants, suggesting a role in encoding taste quality. Later, using a meta-analytic approach, Ponticorvo et al. (2022) showed that sweet taste selectively activated the caudate nucleus, meaning that the meta-analytic findings pointed to a significant functional response to sweet taste stimuli, but not to bitter.

In line with this, Pak et al. (2022) showed that glucose loading increased DA transporter availability in the dorsal striatum, including the caudate nucleus.

Taken together, these findings support the notion that the caudate nucleus is consistently recruited during direct taste perception tasks in physiological conditions.

#### Reward, anticipation and/or motivational component

Complementing the sensory data, we further analyzed paradigms dominated by reward, anticipation, and motivational components, where caudate activity shows the integration of taste signals with top-down factors such as hedonic valuation (pleasantness/unpleasantness) and anticipation. Numerous neuroimaging studies showed that the caudate nucleus plays a central role in the hedonic evaluation of gustatory stimuli and in food-related reward processing. The hedonic system comprises a network of regions that encode the pleasure and motivational value of stimuli. Within this circuitry, the dorsal striatum, and particularly the caudate nucleus, couples these affective signals to goal-directed and habitual behavior. The caudate integrates information about the value of rewards and their emotional relevance, supporting the consolidation of experiences into stimulus–response associations, guiding the shift from deliberate choices to more automatic habits. In this way, hedonic processing emerges as the result of an interplay between emotional, motivational, and procedural mechanisms that converge to direct behavior toward pleasurable outcomes (Balleine et al., 2007; Campos et al., 2022).

In a Positron Emission Tomography (PET) study, Small et al. (2001) showed that cerebral blood flow in the left and right dorsal caudate nucleus during chocolate consumption was positively correlated with pleasantness ratings, suggesting a direct association between caudate activation and subjective hedonic experience. Another following study from the same authors (Small et al., 2003) reported that consumption of a favorite meal elicited DA release in the dorsal caudate, which was associated with experienced pleasure when compared to a fasting state.

Further supporting the role of the caudate in processing palatable foods, Stice et al. (2013) used functional Magnetic Resonance Imaging (fMRI) to compare responses to various high-fat/high-sugar equicaloric chocolate milkshakes and tasteless solutions. The authors found that high-fat content elicited stronger activation in the caudate than high-sugar, suggesting brain activation differences due to a variation in fat and sugar content, regardless of flavor. In female participants, Frankort et al. (2015) observed that visual and oral chocolate stimulation activated the right caudate, and this activation positively correlated with neural predictors of chocolate intake. This activation is not restricted to the hedonic experience during ingestion, as its anticipatory reactivity serves as a critical neural predictor of subsequent short-term eating behavior.

Availability and anticipatory reward also appear to modulate caudate activity. Indeed, Stice and Yokum (2018) demonstrated that an initial intake dose of a high-sugar milkshake increased right caudate activity during the anticipation of additional intake, further implicating this region in expectation-driven reward response.

In addition, other studies have reported individual variability in caudate engagement. Davidenko et al. (2018) showed greater right caudate activation in participants whose taste judgments remained unaffected by conflicting symbolic cues, suggesting a role for the caudate in processing prediction error and in resisting external influences on hedonic evaluation. While this study fits into reward-driven paradigms, it also shows evidence of the caudate's role in sensory fidelity, specifically in individuals who prioritize actual taste perception over external cues.

Thanarajah et al. (2019) provided evidence that post-ingestive signals influence caudate activity. Delayed DA release was observed in the right caudate head after milkshake ingestion, indicating that this region processes gut-derived nutritional feedback and supporting its role in post-ingestive reward evaluation. In addition, subregional changes were associated with the varying individual hedonic responses to sucrose ingestion.

The same year, Frank-Podlech et al. (2019) reported oral stimulation with high- and low- fat yogurt reduced functional connectivity between the caudate nucleus and NST, regardless of fat content. Authors indicate that the caudate nucleus participates in a complex network with homeostatic and reward-related areas, rather than a simple tastants detection.

Moreover, Lender et al. (2020) reported increased left caudate activation in response to images associated with edible stimuli, suggesting a contribution to encoding rewarding gustatory experiences. Finally, complementary evidence from visual stimulation only also supports its hedonic role. Blechert et al. (2016) found greater activation of the left caudate nucleus in response to presentation of high-calorie foods, suggesting its involvement in motivational salience and sensitivity to reward availability. More recently, Skałbania et al. (2025) found, through visual stimulation, that pre-choice activity in the caudate may influence the subsequent stimulus-related reward evaluation, potentially linking reward anticipation to behavioral regulation.

Altogether, these findings underscore the caudate nucleus as a critical subcortical relay station for the integration of taste-related reward signals (also negative ones, such as bitter) encompassing anticipatory, consummatory, and post-ingestive phases of hedonic processing.

#### Food availability and homeostatic modulation

Beyond its role in hedonic evaluation, the caudate nucleus has also been linked to taste perception in relation to physiological states such as hunger and satiety, as well as contextual factors like food availability.

Gautier et al. (1999) reported that the caudate nucleus is involved in the perception of taste after 36 h of fasting, as shown through PET and Magnetic Resonance Imaging (MRI). In another fMRI study, Pelchat et al. (2004) found that under craving-induced conditions, the caudate nucleus exhibited increased activation, particularly on the right side, in participants following a monotonous diet who were imagining liked foods. This supports the role played by the caudate in the neural circuitry of food craving. Jacobson et al. (2010) observed caudate activation in response to oral stimulation during hunger, suggesting striatal involvement in the affective evaluation of taste under motivationally relevant conditions. According to Haase et al. (2011), bilateral caudate activation was more strongly modulated by hunger and satiety in healthy participants when processing reward-related aspects of basic tastants. Lastly, Skałbania et al. (2025) reported that greater pre-choice activity in the caudate nucleus was linked to a higher probability of self-control failure in a subsequent food-choice task.

Taken together, these results highlight the caudate's response to internal states and its role in integrating motivational and contextual cues during gustatory processing.

#### Individual and disease risk factors

Wide variability in caudate nucleus responses to taste-related stimuli has been observed across individuals based on various characteristics, including age, gender, genetic background, and familial disease risk factors.

Regarding both age and gender, evidence suggests their influence on how taste is processed within this nucleus, with variations observed in the modulation of its activation. Jacobson et al. (2010) reported that older adults exhibited more robust and consistent caudate activation in response to gustatory stimuli during hunger, suggesting age-related enhancement of striatal involvement in the affective evaluation of taste under motivationally relevant conditions. On the other hand, Green et al. (2013) found greater caudate activation in young participants compared to middle-aged individuals during the hedonic evaluation of sweet and bitter tastes, revealing a main effect of age. Additionally, Haase et al. (2011) observed that reward processing of some tastants was more strongly modulated by hunger and satiety in males than in females, with bilateral caudate activation.

Dietary habits and disease risk factors also appear to modulate caudate activity. In a study on habitual diet soda consumption, Green and Murphy (2012) found that individuals who consumed greater quantities of diet sodas showed reduced activation in the right caudate head when tasting sweet solutions. Regarding emotional eating, Bohon (Bohon, 2014) showed that in response to milkshake taste receipt, emotional eating scores were negatively related to activation in the bilateral caudate, suggesting a reduced reward in adolescents. Moreover, in a 6-month follow-up study, Gao et al. (2018) found that greater impulsivity in food-related decision-making was associated with reduced spontaneous activity and functional connectivity in the right dorsal caudate, suggesting a diminished reward sensitivity linked to future weight gain. In the same year, Shearrer et al. (2018) reported that repeated exposure to high-fat milkshakes dampened caudate activation, potentially reflecting sensory-specific satiety. In contrast, the same study found that individuals with a familial risk of obesity showed heightened caudate response to palatable taste stimuli. Sadler et al. (2023) further demonstrated that repeated exposure to palatable food cues led to increased caudate activation over time in healthy-weight adolescents, particularly those with a familial risk of obesity, reflecting sustained caudate responsivity as a possible neurobiological marker of vulnerability to future weight gain. Sun et al. (2015) found that individuals carrying a weight gain risk allele exhibited caudate activation during milkshake intake, which was linked to long-term weight gain, suggesting a genotype-dependent role of striatal responsiveness in externally driven eating behavior. Lastly, Horndasch et al. (2016) reported that healthy women with a family history of anorexia nervosa exhibited increased caudate activation during anticipation of both rewarding and aversive chocolate stimuli, indicating heightened striatal responsivity to food-related cues.

Overall, these studies position the caudate nucleus as a dynamic hub for gustatory processing, responsive to individual variability and capable of integrating sensory input with motivational and physiological context.

#### Pharmacological modulation of taste

Pharmacological interventions targeting neurotransmitter systems have been used to investigate the modulation of caudate responses to gustatory stimuli in healthy participants.

Horder et al. (2010) reported that rimonabant, a cannabinoid receptor 1 (CB1) inverse agonist/antagonist that blocks endocannabinoid signaling in striatum and limbic areas, decreased neural responses to aversive stimuli in the right caudate nucleus during visual and oral stimulation with sweet tastants. In another fMRI study combining milkshake tasting and visual stimulation, Murray et al. (2014) found that naltrexone, an antagonist of the opioid receptor μ, decreased reward activation to food in the caudate relative to placebo. In the same year, Tudge et al. (2014) observed that tetrahydrocannabivarin, a CB1 receptor antagonist, increased responses to both aversive and rewarding stimuli in the caudate during visual and oral sweet tastant stimulation. Finally, Dean et al. (2016) showed that bupropion, an antidepressant drug that inhibits the norepinephrine and DA reuptake, elevating its extracellular concentration, increased caudate activity during the anticipation phase of both pleasant and unpleasant cues elicited by visual and oral stimulation with a Belgian chocolate drink. These findings suggest that pharmacological agents can modulate caudate activity, homolaterally and/or bilaterally, during the processing of both rewarding and aversive taste-related stimuli. This is particularly interesting considering that these drugs revealed anti-obesity potential (Makowski et al., 2011; Pi-Sunyer et al., 2006).

### Research on pathological conditions

#### Obesity and metabolic dysfunction

Functional alterations in the dorsal striatum and caudate nucleus have been observed during gustatory information processing through neuroimaging studies on metabolic and eating disorders.

Rothemund et al. (2007) reported that the left caudate body showed increased activation in individuals with obesity, with visual stimulation and independently of hunger or satiation states, suggesting its dopamine-mediated involvement in pathological food-related behaviors. Nummenmaa et al. (2012) observed that obese subjects exhibited diminished responses to appetizing vs. bland food images compared to normal-weight individuals, and this finding was correlated with high glucose metabolism in the dorsal caudate nucleus. Stice et al. (2008a,b) found that female obese adolescents exhibited a reduced caudate activation during milkshake consumption, possibly reflecting lower DA receptor availability and a disrupted striatal response to palatable food intake. Building upon this evidence, Green et al. (2011) observed that in older adults, higher abdominal fat and body mass index (BMI) were associated with reduced caudate activation in response to sucrose, supporting the hypothesis that diminished dopaminergic responsivity in reward circuits may contribute to obesity-related mechanisms. Cornier et al. (2015) reported attenuated neuronal caudate responses to sucrose in women prone to weight gain and obesity compared to men, suggesting a gender-related difference in the striatal processing of sweet taste. Geha et al. (2017) showed that obese individuals exhibited reduced global brain connectivity in the caudate nucleus both at rest and during milkshake consumption, indicating a persistent alteration in striatal network integration associated with obesity. In line with this, Babbs et al. (2013) reported an inverse correlation between BMI and the caudate response to milkshakes, associated with impulsivity but not food reward.

On the other hand, results on metabolic syndrome showed that reduced caudate activation during hedonic evaluation of sucrose under hunger was present in both older adults and individuals with metabolic syndrome, consistent with age-related declines in dopaminergic responsivity within reward circuitry (Jacobson et al., 2017). Moreover, a study from Dunn et al. (2023) reported that weight loss and improved insulin sensitivity in obese individuals presumed to have central insulin resistance, were associated with normalization of taste-related Blood Oxygen Level Dependent (BOLD) fMRI responses, and with less robust changes in the caudate. These results suggest a region-specific modulation of striatal taste perception by metabolic state.

#### Eating disorders

In anorexia nervosa (AN), Wagner et al. (2008) found that compared to healthy controls, individuals recovered from restricting-type AN (involving severe calorie restriction without binging or purging), showed reduced activation in the middle and dorsal caudate in response to nutrient taste, indicating a persistent alteration in striatal processing despite weight restoration. In line with this, Kaye et al. (2020) found that lower caudate activation to tastants during hunger was associated with higher harm avoidance in individuals recovered from AN.

In addition, Cowdrey et al. (2011) reported increased caudate activation in response to aversive taste stimuli among those previously affected by AN, suggesting heightened sensitivity to negative gustatory cues potentially linked to disgust processing. Frank et al. (2018) observed that during a dopamine-related conditioning task, adolescents and young adults with AN showed hyperactivation in the caudate head compared to controls. Then, in the same study, the authors analyzed the prediction error model, the brain's process of comparing expected rewards with actual outcomes, with discrepancies generating signals that help update future expectations and influence reward perception. In this study, they reported elevated prediction error responses in the bilateral caudate head at baseline in individuals with AN. Conversely, in subjects with eating disorders, a greater caudate head response to sucrose receipt was significantly associated with higher reward responsiveness drive scores (Frank et al., 2022, 2023).

Regarding bulimia nervosa (BN), Bohon and Stice (Bohon and Stice, 2012) found that higher negative affect was associated with increased caudate activation during anticipation of a palatable taste. Again, Frank et al. (2013) reported that both BN and recovered AN were associated with reduced dorsal caudate gray matter volume, potentially reflecting structural alterations in dopaminergic reward circuits.

Finally, Stice et al. (2025) reported that lower responsivity of the right caudate to anticipated milkshake tastes, which correlated with feeling fat, predicted the future onset of binge eating or compensatory weight control behaviors over a 4-year follow-up.

#### Alcoholism and depression

Alcohol-related disorders' research reveals multiple mechanisms by which the caudate nucleus participates in the processing of gustatory reward cues, as has been shown in the following fMRI studies. Wilcox et al. (2013) reported that polymorphisms in the α-synuclein gene in heavy alcohol drinkers were significantly associated with caudate activation during exposure to alcoholic beverages, suggesting a link between striatal responsiveness and genetic susceptibility to alcohol-related reward processing.

Ray et al. (2014) studied the G allele, a specific variant of the A118G single-nucleotide polymorphism (SNP) in the OPRM1 gene, which encodes the mu opioid receptor. In this SNP, a nucleotide change from adenine (A) to guanine (G) occurs at a particular location. Authors found that during alcohol cue processing, individuals carrying the G-allele showed stronger negative functional connectivity between the dorsal caudate and key frontal and limbic regions, including the insula and orbitofrontal cortex.

This result suggests altered fronto-striatal integration potentially linked to habitual reward-related behaviors. In the same year, Monnig et al. (2014) observed that in heavy drinkers, reduced white matter integrity was associated with increased caudate activation during alcohol taste cue exposure, indicating that structural disruption in fronto-striatal networks may contribute to altered reward responsivity and impaired control over alcohol consumption. Bidwell et al. (2019) reported that greater dopamine D2 receptor methylation levels were associated with increased caudate activation in response to alcohol cues relative to a non-alcoholic appetitive stimulus, indicating a potential epigenetic modulation of striatal responsivity in alcohol use disorder.

These findings highlight how genetic, structural, and epigenetic factors converge on caudate activation patterns during alcohol taste perception, underscoring its role also in the neurobiology of alcoholism.

Considering depression, fMRI evidence indicates alterations in caudate responsivity to food-related cues. McCabe et al. (2009) reported that unmedicated patients recovered from major depression showed increased bilateral caudate activation in response to aversive food-related stimuli. Furthermore, these patients have a diminished neural response to the potentiation effect produced by the simultaneous presentation of the sight and flavor of the stimuli, both aversive and pleasant conditions. These findings suggest abnormal neural responses to reward that may also involve impairments in the cross-modal integration of sensory stimuli in subjects recovered from depression.

Moreover, it is important to mention that many depressed patients often report perceiving an awful oral taste without eating. A clinical trial conducted by Miller and Naylor (1989) reported an unpleasant taste in a group of 1-week drug-free subjects, and another study showed that some antidepressant drugs can distort gustatory perception (Heath et al., 2006). Indeed, these latter could have an influence on the type of caudate responsivity to food-related cues.

#### Covid-19

Alterations in functional connectivity between the caudate nucleus and cortical areas, as well as gray matter loss in various brain areas, including the caudate nucleus, were found in post-Covid-10 patients, in relation to the cognitive deficit and/or olfactory disorder (Rudroff, 2024; Capelli et al., 2024; Troll et al., 2025). Furthermore, previous histological investigation on the caudate nucleus revealed the angiotensin-converting enzyme 2 (ACE2) expression, implying its possible vulnerability to neuronal SARS-CoV-2 viral damage (Chen et al., 2020). Changes in caudate structure and function have also been observed in a couple of studies on post-Covid-19, assessing both smell and taste function. Schönegger et al. (2020), using MRI, in a few case studies reported that one early post-Covid-19 patient (between 11 and 30 days after onset of disease) presented a symmetric, slightly hyperintense signal in the head of the caudate nucleus, predominantly on the left side. This patient was reported to be hyposmic (i.e., with quantitatively reduced olfactory function) but normogeusic (i.e., with normal gustatory function). No mention of dysgeusia (i.e., qualitative gustatory dysfunction, a distorted perception especially during food ingestion) phenomena was made in this regard. Recently, Cecchini and co-workers, in two groups of post-Covid-19 patients, reported having had or not a chemosensory impairment, including dysgeusia, found a volumetric difference for the right caudate nucleus only, although this difference was not retained after statistical correction, probably due to the small sample size (Cecchini et al., 2024).

## Discussion

The evidence from the literature, encompassing both healthy subjects and pathological conditions, extends the functional *repertoire* of the caudate beyond its classical role, suggesting a broad tuning of this structure also for taste perception. However, drawing a straightforward or definitive interpretation remains challenging, as the findings emerge from studies with heterogeneous methodologies, different participant demographics or clinical backgrounds and various experimental paradigms. In this regard, methodological differences can yield different results, as in the case of Hwang et al. (2019), where the authors emphasize static volumetric associations, while Ponticorvo et al. (2022) show dynamic functional connectivity within a taste-responsive network identified via meta-analysis. Nevertheless, together, these findings complement each other by indicating that the caudate nucleus is structurally involved in processing both sweet and bitter tastes but functionally shows stronger or more consistent activation to sweet tastes. In line with this, while several studies consistently show caudate recruitment during gustatory stimulation and hedonic evaluation, activation patterns vary widely among healthy participants and across pathological conditions. For instance, responses range from caudate activation to palatable foods in healthy participants (Small et al., 2001; Blechert et al., 2016) and hyperactivation in adolescents and young adults with AN (Frank et al., 2018) to blunted activation in obesity and elevated BMI (Stice et al., 2008a,b; Green et al., 2011; Babbs et al., 2013).

Similar difficulties also emerge considering metabolic state and gender. On one hand, a craving-induced condition (Pelchat et al., 2004), hunger (Jacobson et al., 2010), and fasting (Gautier et al., 1999) increase caudate responses in healthy volunteers. On the other hand, caudate activation is modulated by hunger and satiety, and this modulation is also sex-dependent in both healthy (Haase et al., 2011) and obese participants (Cornier et al., 2015). In the latter study, the striatal processing of sweet stimuli brings attention to the role of sweet taste in Parkinson's disease. This is a systemic neurodegenerative disease, with striatal dopamine neuronal progressive depletion, clinically characterized by motor (i.e., tremor, rigidity, bradykinesia, postural instability) as well as various non motor symptoms, among which olfactory deficit is now well documented (Haehner et al., 2011; Postuma et al., 2015; Poewe et al., 2017; Stefani et al., 2021). Regarding taste, previous research found a slight subclinical taste impairment but with sweet taste preservation in time, in patients with advanced stages of disease (Cecchini et al., 2014; Ricatti et al., 2017). More recently taste deficit was suggested to be a prodromal symptom as olfactory deficit and REM sleep behavior disorder are, even if the precise mechanisms are not fully clarified (Alia et al., 2025). Moreover, some studies showed a preference for sweet food, even if the reason for that is not clear yet (Cecchini et al., 2014; Ricatti et al., 2017; Sienkiewicz-Jarosz et al., 2013; Cecchini et al., 2019; Sakamoto et al., 2025). This could be linked to the critical role of the striatum in the complex network of reward, cognition, sensory and motor function (Haber, 2016). Indeed, previous studies reported a heterogeneous striatal distribution of dopamine neuronal depletion in PD (Kish et al., 1988; Wang et al., 2006; Hong et al., 2014) and very recent *post-mortem* research including PD patients' brain samples, found abnormal changes in endoplasmic reticulum (ER) with impaired unfolded protein response in the caudate nucleus of PD compared to controls, impacting on neurotransmission and synaptic vesicle dynamics (Saries-Serrano et al., 2025).

This can support the hypothesis that diminished dopaminergic responsivity in reward circuits may contribute to food-related behaviors and preferences also in this kind of patients (Haase et al., 2011; Sakamoto et al., 2025), even if specific research on the role of the caudate nucleus in this field is needed. Awareness of these findings could be useful in planning patients' diets to reduce the risk of developing metabolic disorders (e.g., diabetes) and the use of artificial sweeteners could be meaningful to improve the pleasure of the meal, bettering the quality of life of these patients (Alia et al., 2025; Jagota et al., 2022). Nevertheless, research on the link between the striatal system and gustation in PD needs to be expanded and the literature's data highlight a promising research direction for this neurodegenerative disorder.

In addition, individual background further shapes caudate function. For instance, genotype-dependent prediction may also modulate caudate responses, as was observed for weight gain (Sun et al., 2015) and AN (Horndasch et al., 2016).

Moreover, age-related differences in caudate activation may explain the discrepancies found among some studies. For instance, Jacobson et al. (2010) found robust caudate activation in older adults, and Green et al. (2013) reported greater activation in younger individuals. First, stimulus type and concentration differed, with Jacobson using broad basic tastes and Green focusing on specific sweet/bitter solutions. Second, task design demands and cognitive load varied from hunger-driven motivational processing in Jacobson to pure hedonic evaluation in Green. Finally, sample size and demographic variability likely contributed, as Green's small, sex-balanced cohort contrasts with Jacobson's larger, age-focused group, potentially affecting the statistical power to detect striatal sensitivity across ages. Furthermore, these discrepancies must be interpreted in the context of neurobiological aging, leading to decreased dopamine function (Dreher et al., 2008), which could alter striatal responses in taste function and reward processing. These changes might explain why older adults in Jacobson et al. (2010) showed robust caudate activation but under specific hunger-driven tasks conditions, while exhibited greater activation during hedonic evaluation tasks in younger adults relative to middle-aged in Green et al. (2013).

In obesity, studies point to both heightened responsivities, independently of the metabolic state (Rothemund et al., 2007), and reduced connectivity in the caudate (Geha et al., 2017). These findings could support the idea of an alteration in the striatal network integration mechanism, which may be causing dysregulation or variable responses. Eating disorders present a similarly complex picture that is difficult to address, with reduced activation in recovered AN patients (Wagner et al., 2008), heightened responses to aversive stimuli (Cowdrey et al., 2011), and structural alterations in caudate volume (Frank et al., 2013), pointing to alterations in dopaminergic reward circuits. Alcoholism studies extend this variability, linking caudate responsivity to genetic (Wilcox et al., 2013), epigenetic (Bidwell et al., 2019), and structural factors (Monnig et al., 2014), while depression is a condition less commonly studied in relation to taste processing, and it has been associated with alteration in the reward system, showing hypersensitivity to negative gustatory cues (McCabe et al., 2009).

Regarding chemosensory research in post-Covid-19 patients, various papers documented the prevalence of persistent symptoms, among which there are olfactory and also gustatory deficit. The latter really is a rarer symptom during the acute infection, while in post-COVID-19 condition was shown as a frequent symptom and suggested to be linked to a decrease central nervous amplification (Cecchini et al., 2022; Hintschich et al., 2024). Indeed, few studies adopted validated psychophysical tests to assess both olfaction and taste, while many of them were based on subjective patients' rating only and sometimes what is reported as taste loss is due to a qualitative disorder (i.e., dysgeusia) and not a quantitative reduction of the gustatory function (i.e., hypogeusia) (Hintschich et al., 2024; Gentilotti et al., 2024; Hintschich et al., 2022). The few interesting results emerged by this literature search on taste, involving the caudate nucleus in this pathological condition, need to be investigated in depth, considering that they are very preliminary and gustatory disorders, particularly dysgeusia, were reported to have a huge impact on the daily life of post-Covid-19 patients (Schönegger et al., 2020; Cecchini et al., 2024). In addition, most studies on chemosensory processing in the context of Covid-19 have predominantly focused on olfactory rather than gustatory dysfunction as well as on cognitive impairment, recently linked to subcortical connectivity alterations (Rudroff, 2024; Troll et al., 2025), leaving the specific contribution of the caudate to taste-related changes less well explored (Gentilotti et al., 2024). Furthermore, it is important to underline that different brain regions are “key regions” both for gustatory and cognitive processing (Mantovani et al., 2024), hence future studies on chemosensation in post-Covid-19 patients, should be include cognitive investigation.

Another intriguing point that emerged from the literature is the occasional involvement of the caudate nucleus from only one hemisphere. Some studies show hemispheric specialization of the gustatory information in humans depending on various factors such as experience, taste quality processing, and handedness, even if the clear ascending pathway is not well established yet (Iannilli and Gudziol, 2019; Faurion et al., 1999; Barry et al., 2001; Stevenson et al., 2013). Insights can also be drawn from olfaction. Brancucci et al. (2009) highlighted that olfactory processing shows significant hemispheric asymmetry, with multiple studies pointing to right-hemisphere dominance in odor perception and discrimination. Specifically, the right nostril often shows an advantage for odor detection and differentiation, reflecting lateralized neural pathways. These findings have been linked to the broader role of the right hemisphere in social and emotional processing, suggesting that sensory asymmetry may extend to chemosensory domains more generally.

Several key aspects emerge from this revision. First, the caudate nucleus consistently appears engaged in taste and reward circuits, regardless of whether activation is observed bilaterally or unilaterally, pointing to possible lateralization effects that warrant further investigation. Second, the involvement of the caudate in both health and disease underscores its role as a hub that integrates gustatory information with motivation, affect, and possible clinical outcomes. Third, another line of evidence highlights the pharmacological modulation of caudate function. Some drugs have been shown to alter caudate responsiveness to rewarding and aversive gustatory cues, underscoring the importance of neurotransmitter systems in shaping striatal involvement in pleasant and unpleasant cues processing. In conclusion, based on information available in current literature, we propose the following functional framework. The caudate nucleus seems to be a critical integrative hub of the striatum, acting as a “interpreter” between basic sensory taste information and motivated behavior. Based on anatomical and neuroimaging literature, caudate behaves as a dynamic and flexible mediator involved both in sensory perception and discrimination but also in reward valuation to gustatory stimuli, influencing action selection within cortico-striatal connections. Hence, it complements taste input with environmental signals and motivational individual state both in health or disease to lead to the appropriate behavior. In that way, it can act as a bridge between sensory perception and motor decision-making plans.

Future research based on big data analysis and functional connectivity approach should clarify the role of the caudate in both normal and pathological gustatory function. For the present literature, what remains undeniable is that the caudate nucleus, the major component of the dorsal striatum, long recognized for its motor and cognitive functions, is also involved in the different facets of the gustatory sensory processing. Its functional involvement in various conditions, modulated by individual traits and drugs, highlights both its flexibility and its possible clinical relevance, providing a foundation for future studies aimed at clarifying its role in the complex network of chemosensation, reward, and disease.
